# Exendin-4 and eldecalcitol synergistically promote osteogenic differentiation of bone marrow mesenchymal stem cells through M2 macrophages polarization via PI3K/AKT pathway

**DOI:** 10.1186/s13287-022-02800-8

**Published:** 2022-03-21

**Authors:** Yupu Lu, Shanshan Liu, Panpan Yang, Yuying Kou, Congshan Li, Hongrui Liu, Minqi Li

**Affiliations:** 1 Department of Bone Metabolism, School and Hospital of Stomatology, Cheeloo College of Medicine, Shandong University and Shandong Key Laboratory of Oral Tissue Regeneration and Shandong Engineering Laboratory for Dental Materials and Oral Tissue Regeneration, Wenhua West Road 44-1, Jinan, 250012 Shandong China; 2 Center of Osteoporosis and Bone Mineral Research, Shandong University, Jinan, 250012 Shandong China

**Keywords:** Exendin-4 (Ex-4), Eldecalcitol (ED-71), Bone marrow mesenchymal stem cells (BMSCs), M2 macrophages, PI3K/AKT pathway

## Abstract

**Background:**

The incidence of diabetic osteoporosis is increasing. This article evaluates the effect of combination treatment with the hypoglycemic drug exendin-4 (Ex-4) and the vitamin D analog eldecalcitol (ED-71) on improving diabetic osteoporosis and explores the relevant mechanism of action.

**Method:**

Micro-CT, HE staining, immunohistochemistry, qPCR and ELISA were used to evaluate the impact of Ex-4 and ED-71 on bone formation and macrophage polarization in a mouse model of diabetic osteoporosis in vivo. Immunofluorescence, flow cytometry and qPCR were used to characterize the polarization type of macrophages treated with Ex-4 and ED-71 in vitro. A co-culture system of BMSCs and macrophages was established. Subsequently, crystal violet staining, alkaline phosphatase staining and alizarin red staining were used to evaluate the migration and osteogenesis differentiation of BMSCs.

**Results:**

Ex-4 combined with ED-71 significantly reduced blood glucose levels and enhanced bone formation in mice with diabetic osteoporosis. In addition, Ex-4 synergized with ED-71 to induce the polarization of macrophages into M2 through the PI3K/AKT pathway. Macrophages treated with the combination of Ex-4 and ED-71 can significantly induce the osteogenic differentiation of BMSCs.

**Conclusion:**

Ex-4 synergized with ED-71 to reduce blood glucose levels significantly. And this combination therapy can synergistically induce osteogenic differentiation of BMSCs by promoting M2 macrophages polarization, thereby improving diabetic osteoporosis. Therefore, the combination of Ex-4 and ED-71 may be a new strategy for the treatment of diabetic osteoporosis.

## Introduction

Diabetic osteoporosis is a serious global public health issue. Both patients with type I diabetes mellitus (T1DM) and those with type II diabetes mellitus (T2DM) may be at increased risk for developing osteoporosis due to the loss of bone mass [1]. Biological therapy for the treatment of diabetic osteoporosis requires a double-pronged approach, since the osteoporosis in question develops in the background of diabetes. Thus, diabetes must also be treated using anti-diabetic therapies so as to limit further deterioration of bone mass [2]. An ideal drug is one that has both hypoglycemic and bone repairing effects; however, there are no such drugs available. Thus, combination therapies represent important pursuits in the treatment of diabetic osteoporosis.

Exendin-4 (exenatide, Ex-4), an analog of glucagon like peptide-1 (GLP-1) [3], improves the function of β-cells and reduces fasting glucose levels, making it one of the most promising treatment methods for patients with T2DM [4]. Another drug, eldecalcitol (1*α*, 25-dihydroxy-2*β*-(3-hydroxypropyloxy) vitamin D_3_; ED-71), is a new type of active vitamin D_3_ analogue that has been approved for the treatment of osteoporosis. An advantage that ED-71 carries relative to traditional vitamin D is that it induces bone mini-modeling [5]. In addition, ED-71 can increase bone mineral density (BMD) more effectively. However, the impact of a combination treatment with both Ex-4 and ED-71 on diabetic osteoporosis remains to be determined, and the related mechanisms of action remain to be explored.

A potential biological mechanism that may influence the treatment of diabetic osteoporosis is the rapidly expanding field of macrophage polarization. For instance, M2 macrophages have been shown to induce the osteogenic differentiation of bone marrow mesenchymal stem cells (BMSCs) and increase bone mineralization by stimulating the release of paracrine cytokines, potentially influencing bone formation patterns in patients with diabetic osteoporosis [6].

In response to environmental signals or pharmacological stimulation, macrophages can be polarized into different phenotypes, which consist of two main subsets: the pro-inflammatory, M1 or classically activated macrophages, and the anti-inflammatory, M2 or alternatively activated macrophages. Several studies have suggested that M2 macrophages may play an important role in the process of osteogenesis. Specifically, M2 macrophages can up-regulate the secretion of transforming growth factor-β1 (TGF-β1), thereby promoting the migration of BMSCs [7]. In addition, M2 macrophages can promote the osteogenic differentiation of BMSCs by secreting bone morphogenetic protein 2 (BMP2), soluble factor inhibitor-M (OSM) and other cytokines [8].

Many pathways are involved in regulating the phenotype and function of macrophages. Notably, phosphatidylinositol 3 kinase/protein kinase B (PI3K/AKT) is one of the most important pathways in the orchestration of metabolic processes in macrophages [9]. After stimulation, the activation of PI3K/AKT pathway can promote polarization to M2 macrophages [10].

This article aimed to explore whether the combination treatment of Ex-4 and ED-71 could promote the osteogenic differentiation of BMSCs through polarization of M2 macrophages. We also sought to verify the connections described regarding the PI3K/AKT signaling pathway, so as to illuminate a new avenue for the treatment of diabetic osteoporosis.

## Materials and methods

### Animals, drug administration and tissue management

Male C57BLKS/J Iar- + Lepr^db^/ + Lepr^db^ mice (db/db) were used as a model of diabetic osteoporosis. Mice (body weight: 33–45 g; age: 8 w; *n* = 4) were obtained from the Institute of Model Animal of Nanjing University (Jiangsu, China) and were kept in standard laboratory conditions with a 12-h light–dark cycle, a constant temperature of 20 °C, and a humidity of 48%. The mice were randomly assigned to four groups: the control group, where mice received PBS orally for 4 weeks; the Ex-4 group, where mice received Ex-4 (10 μg/kg/day, subcutaneously injected) for 4 weeks [11]; the ED-71 group, where mice received ED-71 (0.25 μg/kg, 3 times per week, orally) for 4 weeks [12]; the Ex-4 + ED-71 group, where mice received Ex-4 (10 μg/kg/day, subcutaneously injected) and ED-71 (0.25 μg/kg, 3 times per week, orally) for 4 weeks.

At the end of treatment, the mice were anesthetized and fixed with 4% paraformaldehyde by intracardiac perfusion. After fixation, the femur, tibia and liver were dissected and immersed in the same fixative for 24 h and then first two samples were demineralized with an EDTA solution for 1 month at 4 °C. Then, all specimens were dehydrated through an ascending ethanol series and embedded in paraffin using standard procedures. A series of 4 µm longitudinal sections were prepared for subsequent histological analysis. This study was approved by the Animal Research and Ethics Review Committee of Shandong University Stomatological Hospital (approval number 20210111).

### Micro-CT scan

Following the 4-week treatment, tibias were dissected aseptically. Subsequently, these samples were scanned and three-dimensional images were reconstructed at 10 µm resolution using a micro-CT analysis system (Scanco Medical, Switzerland).

### Hematoxylin–eosin (HE) staining

The sections obtained as described above were dewaxed and rehydrated in preparation for HE staining. The prepared slices were immersed in hematoxylin for 15 min and then washed with distilled water. Then, the slices were stained with eosin for 10 min and washed again. Finally, the stained slices were observed, and digital images were captured with an optical microscope (BX-53, Olympus Corp., Japan).

### Blood glucose measurement and serum biochemical analysis

During the drug interventions, mice in each group were deprived of water for 8 h every 5 days, the tail vein was punctured, and the fasting blood glucose level was measured with a blood glucose meter (Yuwell Medical Equipment Co., Ltd., China).

Blood samples were collected before the db/db mice were sacrificed. Specifically, after the mice were anesthetized, blood samples were collected with the retro-orbital blood sampling method. Serum samples were separated by centrifugation at 3000 rpm for 10 min and stored at − 80 °C. A kit (Century World Biotechnology Co., Ltd., China) was used to detect the level of serum calcium and phosphorus according to the manufacturer’s instructions.

### Immunohistochemical staining

After dewaxing and rehydration, tissue slices were soaked in PBS containing 0.3% hydrogen peroxide for 30 min to inhibit endogenous peroxidase, and blocked with 1% BSA in PBS for 20 min at room temperature to prevent non-specific staining. The treated slices were soaked in their corresponding antibodies for 2 h. The antibodies included rabbit anti-alkaline phosphatase (ALP) (1:250; ab108337, Abcam, UK), rabbit anti-collagen I (1:200; ab34710, Abcam, UK), rabbit anti-CD206 (1:100; ab64693, Abcam, UK) and rabbit anti-TGF-β1 (1:500; ab215715, Abcam, UK). After rinsing with PBS, samples were incubated with horseradish peroxidase-conjugated goat anti-rabbit IgG (1:1000; ab6721, Abcam, UK) for 1 h at room temperature. The immune responses were visualized using diaminobenzidine (Sigma-Aldrich, Merck KGaA, Germany). Subsequently, all slices were re-stained with methyl green and observed under an optical microscope (BX-53, Olympus Corp., Japan).

### Enzyme-linked immunosorbent assay (ELISA)

The polarization of macrophages was identified by measuring serum cytokine levels. The serum was collected from the blood of administered mice and control mice. The levels of IL-1β and TGF-β1 were measured using a cytokine-specific ELISA kit (Proteintech, USA), according to the manufacturer’s instructions.

The secretion of TGF-β1 was measured in vitro. The supernatants from cells of the CON group and the drug administration group were collected. The level of TGF-β1 was measured using a cytokine-specific enzyme-linked immunosorbent assay (ELISA) kit (Proteintech, USA), according to the manufacturer’s instructions.

### Cell culture

RAW264.7 cells were obtained from Shanghai Cell Center (Shanghai, China). BMSCs were isolated from the tibia and femur of 8-week-old C57BL/6 male mice. Specifically, the tibia and femur were aseptically dissected after cervical vertebra dislocation of a mouse, and the proximal and distal ends of the bones were removed. Then, the exposed bone marrow was flushed into a culture flask with α-modified Eagle’s medium (α-MEM) containing 20% fetal bovine serum (FBS). After a 5–7-day incubation, the medium was replaced. According to the manufacturer's protocol, flow cytometry (FACS Verse 8, Becton Dickinson, New York, USA) was used to identify the phenotypic surface biomarkers (positive biomarkers: CD73 and CD105; negative biomarkers: CD34 and CD45) of BMSCs. Subsequently, these two kinds of pure cells were collected in high glucose-Dulbecco’s MEM (H-DMEM) or α-MEM containing 10% FBS, 100 U/mL penicillin and 100 mg/mL streptomycin and maintained at 37 °C in a 5% CO_2_ humidified incubator.

RAW264.7 cells were transferred to serum-free medium 12 h before treatment. The medium was then replaced with medium containing 1 × 10^–8^ M Ex-4 (HY-13443, MedChemExpress, USA) [13], 1 × 10^–9^ M ED-71 (Chugai Pharmaceutical Co., Ltd., Japan) [14], or 1 × 10^–8^ M Ex-4 and 1 × 10^–9^ M ED-71, followed by incubation for 48 h. In the inhibition study, cells were pretreated for 24 h with 10 μM LY294002 (HY-10108, MedChemExpress, USA) [15] or 1 μM ARQ092 (HY-19719, MedChemExpress, USA) [15]. After the 24 h pretreatment, Ex-4 with or without ED-71 were added to the cells for 48 h. Finally, they were harvested for immunofluorescence, flow cytometry, quantitative real-time PCR (qPCR), western blotting and ELISA analyses.

To evaluate the migration of BMSCs in the co-culture system, BMSCs were cultured in the upper chamber and RAW264.7 cells were seeded in the lower chamber. To investigate the osteogenesis differentiation of BMSCs, RAW264.7 cells were cultured in the upper chamber and BMSCs were seeded in the lower chamber. The process of administration of Ex-4 and ED-71 to RAW264.7 cells was performed as described above. RAW264.7 cells were transferred to serum-free medium for 12 h prior to treatment, and then co-cultivation was performed. Subsequently, the medium of RAW264.7 cells was replaced with media containing the corresponding concentration of Ex-4 with or without ED-71, and the system was incubated for 48 h. Inhibition studies were also performed, where RAW264.7 cells were pre-treated with 10 μM LY294002 or 1 μM ARQ092 for 24 h before adding Ex-4 with or without ED-71. Cells were analyzed with crystal violet staining, alkaline phosphatase (ALP) staining, and alizarin red (AR) staining.

### Immunofluorescence (IF) analysis

Cells were fixed with 4% paraformaldehyde for 20 min. Then, they were blocked with 5% BSA in PBS for 1 h and incubated with the following specific antibodies at 37 °C for 1 h. The primary antibodies were rabbit anti-CD163 (1:300; ab182422, Abcam, UK) and mouse anti-CD206 (1:300; 60143-1-Ig, Proteintech, USA). Subsequently, cells were then incubated with goat anti-rabbit IgG H&L (Alexa Fluor® 488) (1:200; ab150081, Abcam, USA) and goat anti-mouse IgG H&L (Alexa Fluor® 647) (1:200; ab150119, Abcam, USA) for 1 h. Nuclei were stained with DAPI, and images were obtained by fluorescence microscopy (Model DMi8 automated, Leica Microsystems CMS GmbH, Germany).

### Flow cytometry

Flow cytometry was performed according to the instructions of the manufacturer of the flow cytometer. Briefly, a suspension of RAW264.7 cells was prepared in 0.1% BSA in PBS and centrifuged at 300*g* for 5 min, and the supernatant was discarded. In order to reduce non-specific staining during the staining process, the suspension was incubated with 0.5 μg purified anti-mouse CD16/32 antibody [2.4G2] (E-AB-F0997A, Elabscience, China) for 10 min. F4/80-FITC (ab105155, Abcam, UK) and CD206-PE (ab223906, Abcam, UK) were used to incubate at 4 °C in the dark for 60 min. Then, 5 mL 0.1% BSA in PBS was added to resuspend cells and centrifuged at 300*g* for 5 min. The supernatant was discarded. Repeat washing 2 times. Finally, 0.5 mL 0.1% BSA in PBS was added to resuspend cells, and the polarization types of macrophages were identified by flow cytometer (BD Accuri C6 Plus, Becton, Dickinson and Company, USA).

According to the manufacturer's protocol, BMSCs were digested with trypsin, and the digestion was terminated at an appropriate time. Centrifugation was performed at 2000 rpm for 5 min. Then, the supernatant was discarded and the cells were resuspended in 100 μL 0.1% BSA in PBS. The cells were incubated with CD34-FITC (553733, BD, USA), CD45-PE (561087, BD, USA), CD73-PE (550741, BD, USA) and CD105-FITC (ab221675, Abcam, UK) at 4 °C in the dark for 60 min. 5 mL 0.1% BSA in PBS was added to resuspend cells and centrifuged at 300*g* for 5 min. Subsequently, the supernatant was discarded after centrifugation at 300*g* for 5 min, and the samples were washed twice. Finally, 500 μL 0.1% BSA in PBS was used to resuspend the cells again. The purity of BMSCs was identified by flow cytometer as above.

### RNA isolation and quantitative real-time PCR (qPCR)

For the isolation of RNA from bone, the bones of sacrificed mice with cervical dislocation were ground quickly. During this period, liquid nitrogen was continuously added to prevent the sample from melting until the sample became powder. For RNA isolation from RAW264.7 cells, the medium was discarded, and cells were rinsed 3 times with precooled PBS. Subsequently, Trizol reagent (AG21102, Precision Biotechnology (Human) Co., Ltd., China) was used to extract RNA from bone samples and RAW264.7 cells. According to the manufacturer's protocol, cDNA was synthesized by using the Evo *M-MLV* RT Reverse Transcription kit II (AG11711, Accurate Biotechnology (Human) Co., Ltd., China). SYBR Green Pro Taq HS premixed qPCR kit (AG11701, Accurate Biotechnology (Human) Co., Ltd., China) was used to perform qPCR in real-time fluorescence quantitative PCR system (LightCycler® 96 SW 1.1, Roche Ltd, Switzerland). The primer sequences are shown in Table 1. All data were normalized to GAPDH expression, and quantification of qPCR results was performed by the 2^−∆∆CT^ method.

**Table 1:** Primer sequences for reverse transcription quantitative PCR

cDNA	Upstream primer	Downstream primer
IL-1β	F: 5′-TCCAGGATGAGGACATGAGCAC-3′	R: 5′-GAACGTCACACACCAGCAGGTTA-3′
iNOS	F: 5′-CAAGCTGAACTTGAGCGAGGA-3′	R: 5′-TTTACTCAGTGCCAGAAGCTGGA-3′
CD163	F: 5′-ACTTCAGAATCACATCATGGCACA-3′	R: 5′-TCGTCGCTTCAGAGTCCACAG-3′
CD206	F: 5′-AGAGCTGGCGAGCATCAAGAG-3′	R: 5′-TTCCATAGGTCAGTCCCAACCAA-3′
Nos2	F: 5′-CCTGCTTTGTGCGAAGTGTC-3′	R: 5′-CCCAAACACCAAGCTCATGC-3′
Ptgs2	F: 5′-AGCCAGGCAGCAAATCCTT-3′	R: 5′-GGGTGGGCTTCAGCAGTAAT-3′
TNF-α	F: 5′- CGGGCAGGTTCTACTTTGGAG-3′	R: 5′-ACCCTGAGCCATAATCCCCT-3′
Arg1	F: 5′-AGACCACAGTCTGGCAGTTG-3′	R: 5′-TGTCAGTGTGAGCATCCACC-3′
Chil3	F: 5′-TGTTCTGGTGAAGGAAATGCGTA-3′	R: 5′-AGTAGCAGCCTTGGAATGTCTT-3′
Retnla	F: 5′-CCCTTCTCATCTGCATCTCCC-3′	R: 5′-AGTGGAGGGATAGTTAGCTGG-3′
TGF-β1	F: 5′-AGGGCTACCATGCCAACTTC-3′	R: 5′-CCACGTAGTAGACGATGGGC-3′
GAPDH	F: 5′-GCACCGTCAAGGCTGAGAAC-3′	R: 5′-TGGTGAAGACGCCAGTGGA-3′

### Crystal violet staining, alkaline phosphatase (ALP) staining, and alizarin red (AR) staining

The co-cultured BMSCs were fixed with 4% paraformaldehyde for 20 min and then stained with crystal violet for 15 min to detect the migration of BMSCs. Areas of interest were selected under the optical microscope (CKX-41, Olympus Corp., Japan).

To evaluate the ALP activity of BMSCs, the co-cultured BMSCs were fixed with 4% paraformaldehyde for 15 min and stained with ALP solution according to the manufacturer's protocol. Imaging was obtained through an optical microscope (CKX-41, Olympus Corp., Japan). To evaluate the deposited minerals, the co-cultured BMSCs were fixed with 4% paraformaldehyde for 15 min and then stained with 1% AR staining solution (Lot. No.20180820, Solarbio, China) to detect the degree of mineralization. Subsequently, AR was further isolated with cetylpyridinium chloride and was detected using a spectrophotometer (iMark, Bio-Rad Laboratories, Inc., USA).

### Western blotting

RIPA Lysis Buffer (Lot 02408/60412, CwBio Biotechnology Co., Ltd., China) was used to extract proteins according to the manufacturer's instructions. The protein concentrations were determined by using the BCA protein concentration assay kit (P0012S, BeyoTime Biotechnology, China). The protein samples were mixed with 1/4 volume of 5 × SDS loading buffer and heated at 95 °C for 5 min. Equal amounts of proteins were separated by 10% SDS-PAGE gel electrophoresis and transferred onto a polyvinylidene fluoride (PVDF) membrane. After blocking with 5% BSA-TBST at room temperature for 1 h, the membranes were incubated overnight at 4 °C with the following primary antibodies: rabbit anti-p-PI3K (1:1000; ab278545, Abcam, UK), rabbit anti-p-AKT (1:1000; ab192623, Abcam, UK), rabbit anti-β-catenin (1:4000; ab6302, Abcam, UK) and rabbit anti-GAPDH (1:2500; ab9485, Abcam, UK). Samples were then incubated with horseradish peroxidase-conjugated goat anti-rabbit IgG (1:2000; ab6721, Abcam, UK) for 1 h. Enhanced chemiluminescence reagent (B500024, Proteintech, USA) was used to detect immunoreactive bands. Images were captured using a gel imaging system (Amersham Imager 600, General Electric Company, USA).

### Statistical analysis

The data were presented as mean values ± standard deviation and contained at least three independent biological copies. Statistical analyses were conducted with GraphPad Prism 6.0 software (GraphPad Software, Inc., USA). The differences between two groups were identified by Student’s *t*-tests. The differences between three or more groups were tested by one-way analysis of variance (ANOVA). Differences for which *p* < *0.05* were considered significant.

## Results

### Ex-4 synergizes with ED-71 to stimulate bone formation and reduce blood glucose levels in db/db mice

**Fig. 1:**
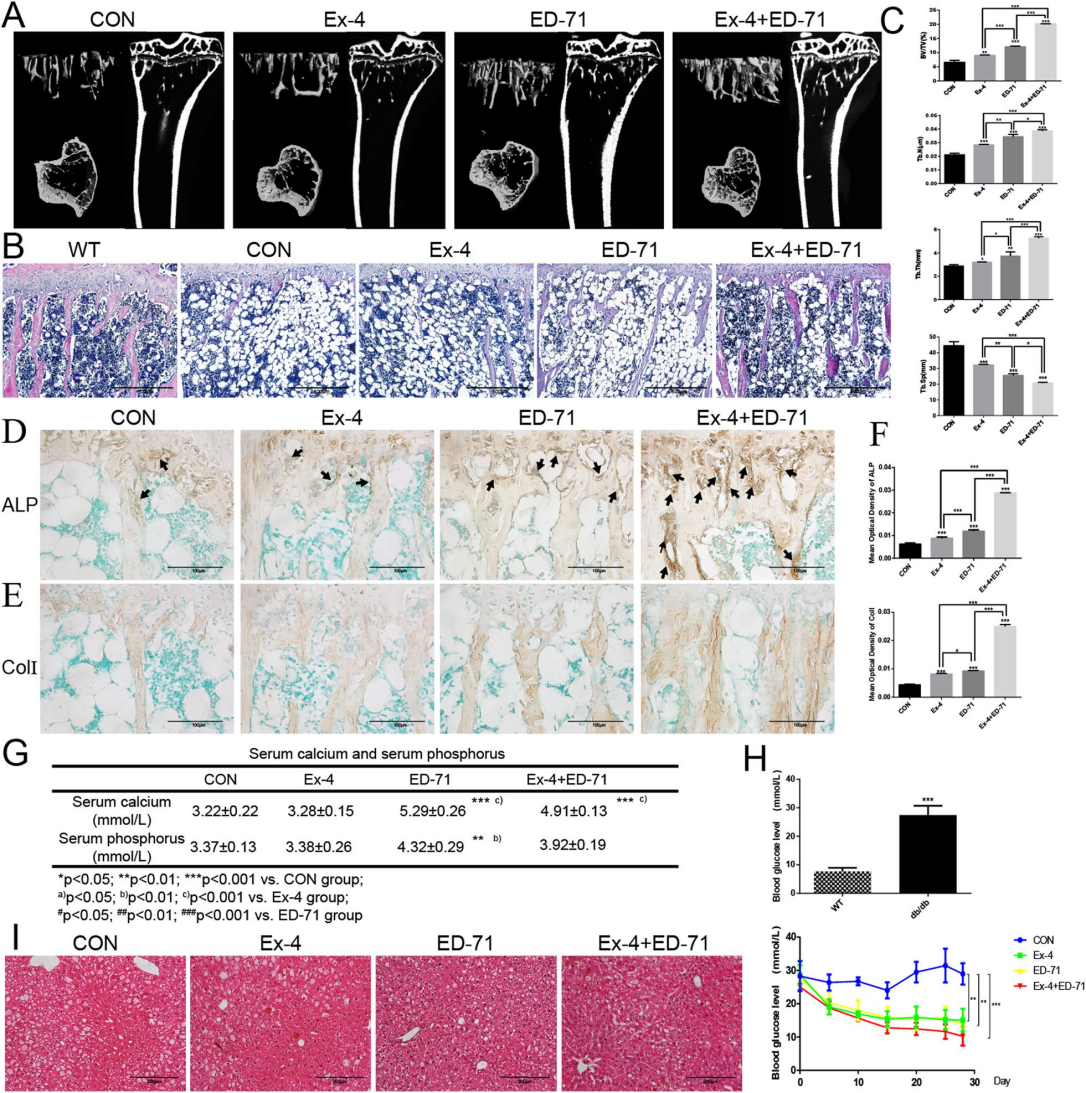
Ex-4 synergizes with ED-71 to stimulate bone formation and reduce the blood glucose levels in db/db mice. **A** Typical three-dimensional and two-dimensional coronal images of tibia were obtained by Micro-CT. **B** HE staining. Bar, 500 μm. **C** The bone volume/tissue volume, trabeculae bone number, trabeculae bone thickness and trabeculae bone space were further analyzed in HE staining (*n* = 4). **D** and **E** Immunohistochemistry of ALP (arrows) (**D**) and immunohistochemistry of Col I (brown color) (**E**). Bar, 100 μm. **F** Immunoreactivity quantitative evaluation of ALP and Col I. **G** Serum calcium and phosphorus levels (*n* = 4). **H** Comparison of blood glucose levels between WT and db/db mice, and changes in blood glucose of each group during the treatment (*n* = 4). **I** HE staining. Bar, 200 μm. Values are expressed as mean ± standard deviation, *p < 0.05, **p < 0.01, ***p < 0.001. *CON* control, *Ex-4* exendin-4, *ED-71* eldecalcitol, *Ex-4* + *ED-71* exendin-4 + eldecalcitol, *WT* wild-type mice, *BV/TV* bone volume/tissue volume, *Tb.N* trabeculae bone number, *Tb.Th* trabeculae bone thickness, *Tb.Sp* Trabeculae bone space, *ALP* alkaline phosphatase, *Col I* collagen I

The effect of Ex-4 and ED-71 on bone formation was investigated in db/db mice. Micro-CT showed that bone formation was significantly increased in db/db mice treated with Ex-4 or ED-71, especially in mice given the combination of Ex-4 and ED-71 (Fig. 1A). Compared with wild-type mice, db/db mice have more vacuoles in the medullary cavity and fewer trabecular bones, but the vacuoles were significantly reduced, and the number of trabecular bones were increased slightly in the group treated with Ex-4. In the group treated with ED-71, the number of vacuoles did not change significantly, but the number of trabecular bones were increased significantly. Moreover, the combination of Ex-4 and ED-71 not only reduced vacuoles, but also increased the number of trabecular bones significantly (Fig. 1B). Compared with CON group, the ratio of bone volume to tissue volume (BV/TV), number of trabecular bones (Tb.N), and thickness of trabecular bones (Tb.Th) were increased, and the trabecular bone space (Tb.Sp) was decreased after administration of both Ex-4 and ED-71. Moreover, the results also showed that ED-71 was more potent than was Ex-4, and the effect of the combined application of the two drugs was significantly stronger than that of either single drug (Fig. 1C). As expected, the immunohistochemical results showed that the combined administration of Ex-4 and ED-71 significantly promoted bone formation in db/db mice. Specifically, Ex-4 or ED-71 increased the immunoreactivity of ALP (Fig. 1D) as well as the immunoreactivity of col I (Fig. 1E). The combined treatment with Ex-4 and ED-71 also appeared to have a greater potency to maintain osteoblast function than Ex-4 or ED-71, as indicated by more intense immunostaining for ALP and col I. Collectively, these results suggest that combined treatment with Ex-4 and ED-71 can increase bone formation significantly in db/db mice. In addition, ED-71 and the combination of Ex-4 and ED-7 significantly increased blood calcium and phosphorus levels, but these differences were not statistically significant (Fig. 1G). While db/db mice have abnormally high blood glucose levels compared with wild-type mice, it was found that ED-71 also had a hypoglycemic effect during the treatment. Additionally, the hypoglycemic effect of the combination treatment was stronger than that of monotherapy (Fig. 1H). Furthermore, HE staining showed that there was no significant difference in liver morphology between the administration group and the CON group, except for the reduction of micro-vesicular steatosis in the liver after administration (Fig. 1I).

### Ex-4 and ED-71 synergistically promote M2 macrophage polarization in vivo and in vitro

**Fig. 2:**
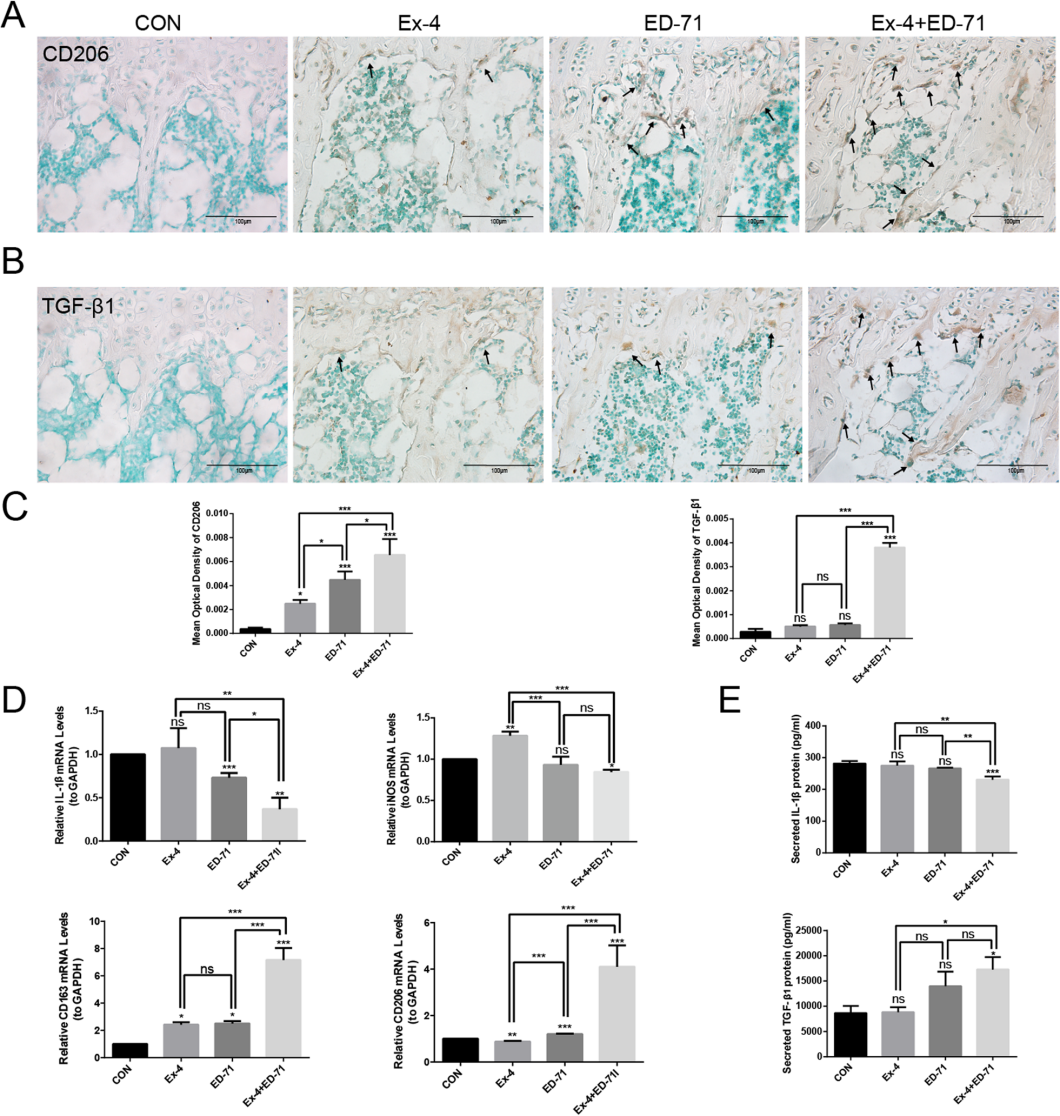
Ex-4 and ED-71 synergistically promote M2 macrophages polarization in vivo. **A** Immunohistochemistry of CD206. Bar, 100 μm. **B** Immunohistochemistry of TGF-β1. Bar, 100 μm. **C** Immunohistochemical quantitative analysis of CD206 and TGF-β1 (*n* = 4). **D** The mRNA expression levels of macrophages marker genes (M1 macrophage: IL-1β and iNOS; M2 macrophage: CD163 and CD206) in the bone tissue from db/db mice (*n* = 3). **E** ELISA was used to detect the secretion cytokine IL-1β of M1 macrophages and the secretion cytokine TGF-β1 of M2 macrophages in serum (*n* = 3). *p < 0.05, **p < 0.01, ***p < 0.001. *CON* control, *Ex-4* exendin-4, *ED-71* eldecalcitol, *Ex-4* + *ED-71* exendin-4 + eldecalcitol, *TGF-β1* transforming growth factor-β1, *IL-1β* interleukin-1β, *iNOS* inducible nitric oxide synthase

The effect of drug administration on macrophage polarization during bone formation was explored by immunohistochemistry. As shown in figure, the immunoreactivity of the M2 biomarker CD206 was significantly increased upon treatment with Ex-4 and ED-71. However, the immunoreactivity of CD206 in either the Ex-4 or ED-71 group was not as strong as that upon combination treatment (Fig. 2A). The combined administration of Ex-4 and ED-71 also increased the immunoreactivity of TGF-β1 significantly. Although the immunoreactivity of TGF-β1 was slightly increased by Ex-4 or ED-71 monotreatment, there was no statistically significant difference compared with the CON group (Fig. 2B). These data suggested that Ex-4 and ED-71 synergistically promote M2 macrophage polarization. Analysis of mRNA expression further revealed that the expression of M1 macrophage biomarkers IL-1β and iNOS decreased and M2 macrophage biomarkers CD163 and CD206 increased in db/db mice treated with co-administration compared to the CON group (Fig. 2D). The secretion of cytokines into the serum of mice was detected by ELISA, and the levels of pro-inflammatory factor IL-1β secreted by M1 macrophages were found to be higher in the CON group and lower in the combined group. Conversely, the levels of the M2 macrophage secreted anti-inflammatory factor TGF-β1 were lower in the CON group and higher in the combination group (Fig. 2E). In summary, the results suggested that Ex-4 and ED-71 can synergically increase macrophage polarization towards M2.

**Fig. 3:**
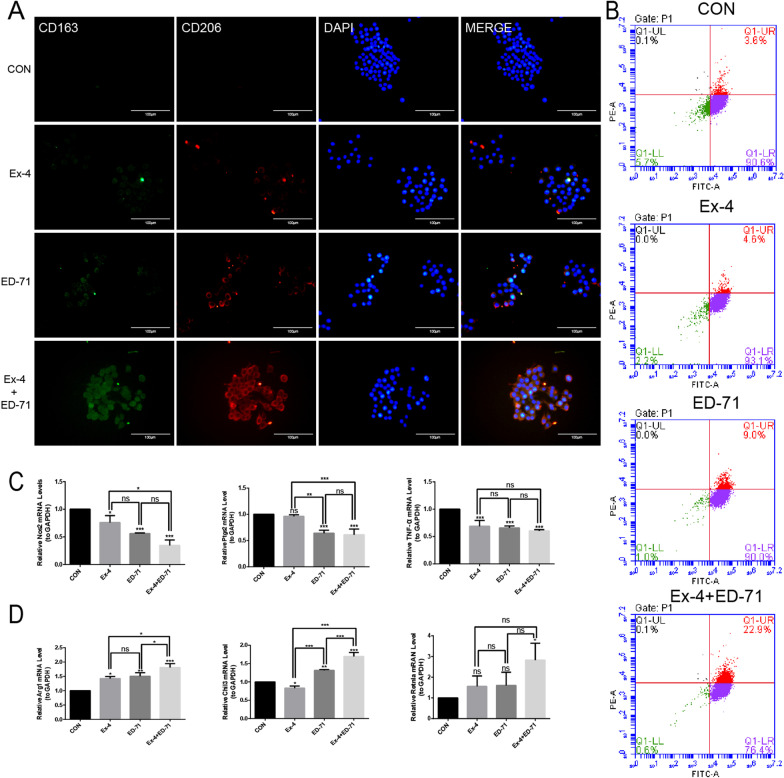
Ex-4 synergizes with ED-71 to increase the proportion of M2 macrophages in vitro. **A** Immunofluorescence staining of CD163 (green) and CD206 (red) in CON, Ex-4, ED-71 and Ex-4 + ED-71 group. The nucleus was counterstained with DAPI (blue). Bar, 100 μm. **B** Flow cytometry was used to analyze the polarization of M2 macrophages in different groups. **C** and **D** The relative mRNA levels of M1 macrophages biomarkers (Nos2, Ptgs2, TNF-α) (**C**) and M2 macrophages biomarkers (Arg1, Chil3, Retnla) (**D**) were detected by qPCR after the administration of Ex-4 and ED-71 (*n* = 3). *p < 0.05, **p < 0.01, ***p < 0.001. *CON* control, *Ex-4* exendin-4, *ED-71* eldecalcitol, *Ex-4* + *ED-71* exendin-4 + eldecalcitol

The polarization of macrophages after the administration of Ex-4 and ED-71 was further analyzed in vitro. In immunofluorescence studies, a relatively weak green (CD163) and red (CD206) fluorescence intensity was observed in Ex-4 group, a much stronger green (CD163) and red (CD206) fluorescence was detected in ED-71 group, while the strongest green (CD163) and red (CD206) fluorescence was detected in the combination group (Fig. 3A). The effect of Ex-4 and ED-71 on the polarization of macrophages was also analyzed by flow cytometry. In the CON group, the proportions of F4/80^+^CD206^+^ cells were very low (3.6%). However, after the treatment with Ex-4 or ED-71, the proportions of F4/80^+^CD206^+^ cells were increased slightly. Specifically, the ratio of F4/80^+^CD206^+^ cells reached 4.6% after the administration of Ex-4, and it increased to 9.0% after ED-71 administration. Furthermore, the proportions of F4/80^+^CD206^+^ cells in the combination group increased to 22.9% (Fig. 3B). This further suggested that Ex-4 and ED-71 can induce M2 macrophages polarization synergistically. As displayed in figure, mRNA expression levels of the M1 macrophage biomarkers Nos2, Ptgs2, and TNF-α were significantly decreased, and those of the M2 macrophages biomarkers Arg1, Chil3, and Retnla were significantly increased in the combination treatment group relative to control (Fig. 3C, D). In conclusion, Ex-4 synergized with ED-71 to promote the polarization of macrophages into M2.

### Macrophages treated with the combination of Ex-4 and ED-71 promote the osteogenic differentiation of BMSCs

**Fig. 4:**
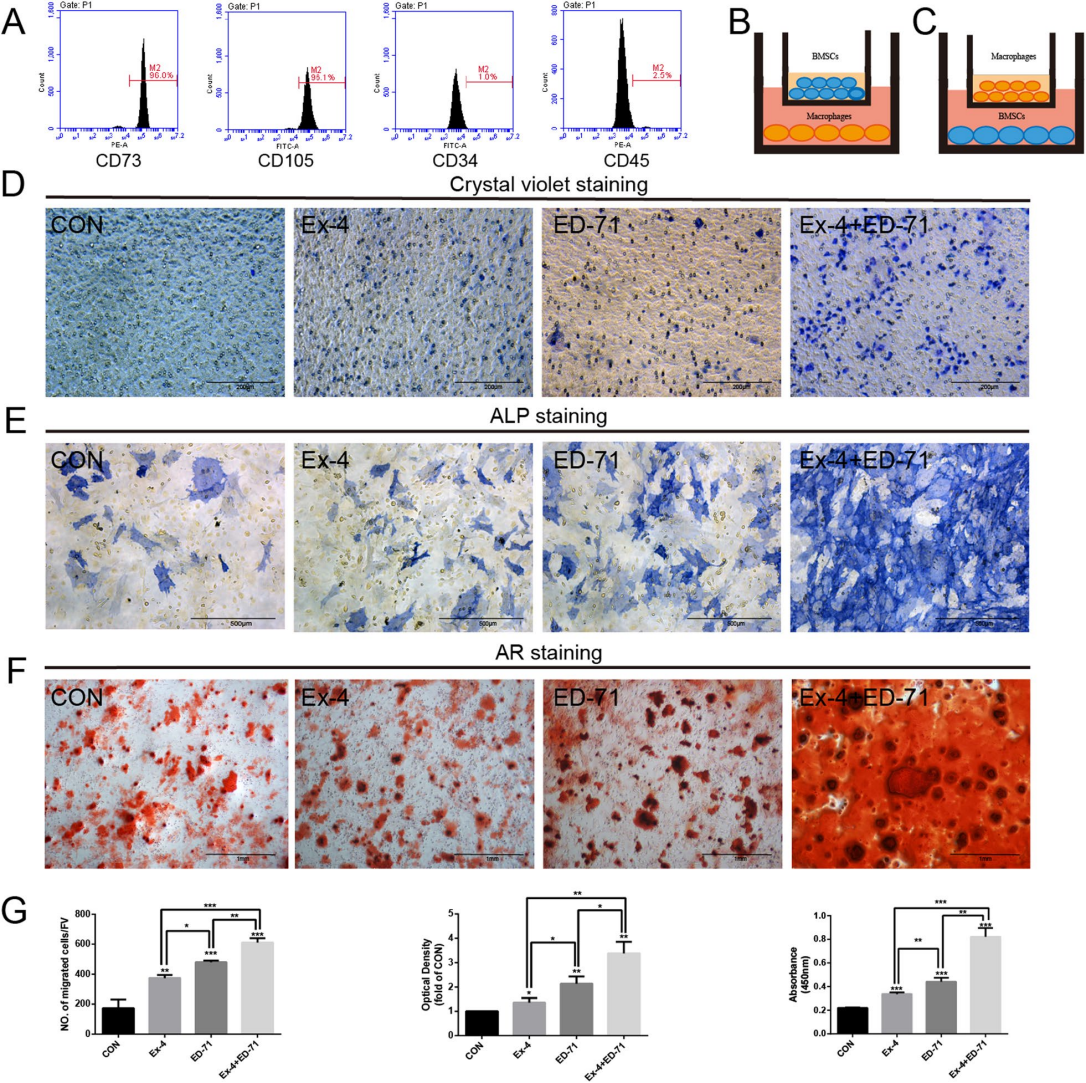
Macrophages treated with the combination of Ex-4 and ED-71 promote the osteogenic differentiation of BMSCs. **A** Identification of BMSCs by flow cytometry. CD73 and CD105 were positive, while CD34 and CD45 were negative. **B** Pattern of co-culture system. BMSCs were inoculated in the upper chamber and RAW264.7 cells were cultured in the lower chamber. **C** Pattern of co-culture system. RAW264.7 cells were inoculated in the upper chamber and BMSCs were cultured in the lower chamber. **D** Crystal violet staining. Using the pattern of co-culture system "BMSCs up", it was found that Ex-4 and ED-71 promote the migration of BMSCs by acting on macrophages. Bar, 200 μm. **E** ALP staining. Using the pattern of co-culture system "RAW264.7 up", it was illustrated that Ex-4 and ED-71 promoted the osteogenic differentiation of BMSCs by acting on macrophages. Bar, 500 μm. **F** AR staining. Using the pattern of co-culture system "RAW264.7 up", it was shown that Ex-4 and ED-71 promoted the osteogenic differentiation of BMSCs by acting on macrophages. Bar, 1 mm. **G** The results of crystal violet staining, ALP staining and AR staining were quantitatively evaluated. *p < 0.05, **p < 0.01, ***p < 0.001. *CON* control, *Ex-4* exendin-4, *ED-71* eldecalcitol, *Ex-4* + *ED-71* exendin-4 + eldecalcitol, *BMSCs* bone marrow mesenchymal stem cells osteogenesis, *ALP* alkaline phosphatase, *AR* alizarin red

BMSCs were obtained and verified for negative expression of surface biomarkers CD34 and CD45 and positive expression of CD73 and CD105 (Fig. 4A). Transwell assays were performed to confirm the effect of RAW264.7 cells treated with Ex-4 and ED-71 on BMSCs. As shown in figure, a co-culture system was used in which the isolated BMSCs were inoculated in the upper chamber and RAW264.7 cells were cultured in the lower chamber (Fig. 4B). Compared to the CON group, RAW264.7 cells treated with Ex-4 or ED-71 altered the migration ability of BMSCs. Moreover, RAW264.7 cells treated with the combination of Ex-4 and ED-71 enhanced the migration ability of BMSCs to the greatest extent (Fig. 4D). Furthermore, in another co-culture system, RAW264.7 cells were inoculated in the upper chamber and BMSCs were inoculated in the lower chamber (Fig. 4C). The results of ALP staining revealed that RAW264.7 cells treated with Ex-4 or ED-71 alone partially enhanced the osteogenic differentiation of BMSCs. In addition, the positive region of the combination treatment group was the most obvious, suggesting that the combination of these two drugs could synergically promote the osteogenic differentiation of BMSCs through RAW264.7 cells (Fig. 4E). Consistently, the results of AR staining illustrated that Ex-4 or ED-71 stimulation alone increased the area of mineralized nodules compared with CON group, while co-treatment further enhanced this phenomenon significantly (Fig. 4F). These results suggested that Ex-4 and ED-71 can synergistically promote osteogenic differentiation of BMSCs through macrophages.

### Ex-4 synergizes with ED-71 to have a potential regulatory effect on macrophages via the PI3K/AKT pathway

**Fig. 5:**
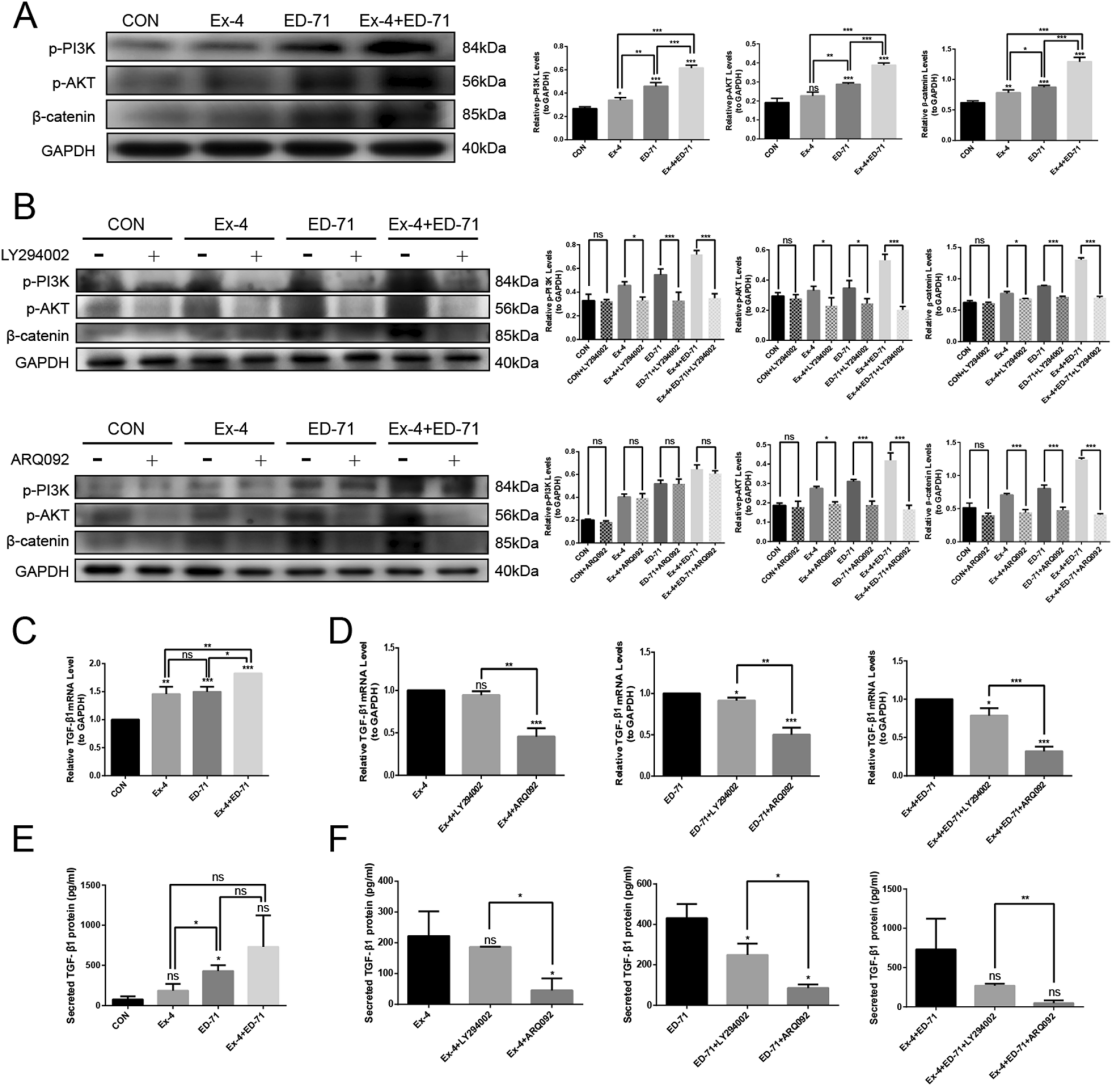
Ex-4 synergizes with ED-71 to have a potential regulatory effect on macrophages via PI3K/AKT pathway. **A** Western blotting was used to detect the protein expression levels of p-PI3K, p-AKT, β-catenin in macrophages of each group, then quantitative analysis was carried on. **B** Macrophages in each group were treated with LY294002(PI3K inhibitor) and ARQ092 (AKT inhibitor), the protein expression levels of p-PI3K, p-AKT and β-catenin were detected by western blotting. Then the quantitative analysis was performed. **C** The relative mRNA levels of TGF-β1 in macrophages of each group were detected by qPCR. **D** The relative mRNA levels of TGF-β1 in each group after the addition of LY294002 or ARQ092. **E** The secretion of TGF-β1 in macrophages of each group was detected by ELISA. **F** The secretion of TGF-β1 in each group after the addition of LY294002 or ARQ092. *p < 0.05, **p < 0.01, ***p < 0.001. *CON* control, *Ex-4* exendin-4, *ED-71* eldecalcitol, *Ex-4* + *ED-71* exendin-4 + eldecalcitol, *p-PI3K* phosphorylation-phosphatidylinositol 3-kinase; *p-AKT* phosphorylation-protein kinase B, *GAPDH* glyceraldehyde-3-phosphate dehydrogenase, *TGF-β1* transforming growth factor-β1

The results of previous experiments in this study confirm that Ex-4 and ED-71 synergistically promote polarization of M2 macrophages. The activation of PI3K/AKT signaling has been considered to be an important step towards this polarization. Thus, we aimed to investigate the relationship between the mechanism of Ex-4 combined with ED-71 on M2 macrophages and the PI3K/AKT pathway. Here, we found that treatment with Ex-4 or ED-71 alone increased the levels of phosphorylated PI3K and AKT and that combined treatment with Ex-4 and ED-71 resulted in a greater increase of phosphorylation. Because previous studies have shown that PI3K/AKT stabilizes β-catenin and promotes its transfer to the nucleus for gene transcription, we further analyzed expression of this downstream target. Indeed, we confirmed that increased expression of β-catenin was most pronounced upon combination treatment group, while the expression of β-catenin was enhanced slightly in Ex-4 and ED-71 monotreatment groups relative to control (Fig. 5A). To further confirm that PI3K/AKT pathway is involved in M2 macrophage polarization mediated by Ex-4 and ED-71, small molecule inhibitors targeting PI3K (LY294002) or AKT (ARQ092) were used. As shown in the figure, p-PI3K and p-AKT was inhibited by LY294002 and ARQ092. Treatment with LY294002 and ARQ092 significantly inhibited the increase in the expression of β-catenin caused by co-treatment with Ex-4 and ED-71, as indicated by the weaker band seen in Figure (Fig. 5B). These results indicated that PI3K/AKT may play a role in the mechanism of action of Ex-4 and ED-71, at least in the upstream signaling of β-catenin in RAW264.7 cells. TGF-β1, which is a cytokine produced by M2 macrophages, is considered to be one of the key factors in the promotion of migration and osteogenic differentiation of BMSCs. The results of qPCR and ELISA showed that the levels of TGF-β1 varied among the different groups. Combination treatment with Ex-4 and ED-71 significantly increased TGF-β1 expression and secretion compared with either monotherapy (Fig. 5C, E). The expression and secretion of TGF-β1 was significantly inhibited by the addition of LY294002 or ARQ092 in the administration groups (Fig. 5D, F). These results indicated that Ex-4 and ED-71 could synergistically promote M2 macrophage polarization through the PI3K/AKT pathway.

### The combination of Ex-4 and ED-71 regulates macrophages through PI3K/AKT signaling pathway to promote osteogenic differentiation of BMSCs

**Fig. 6:**
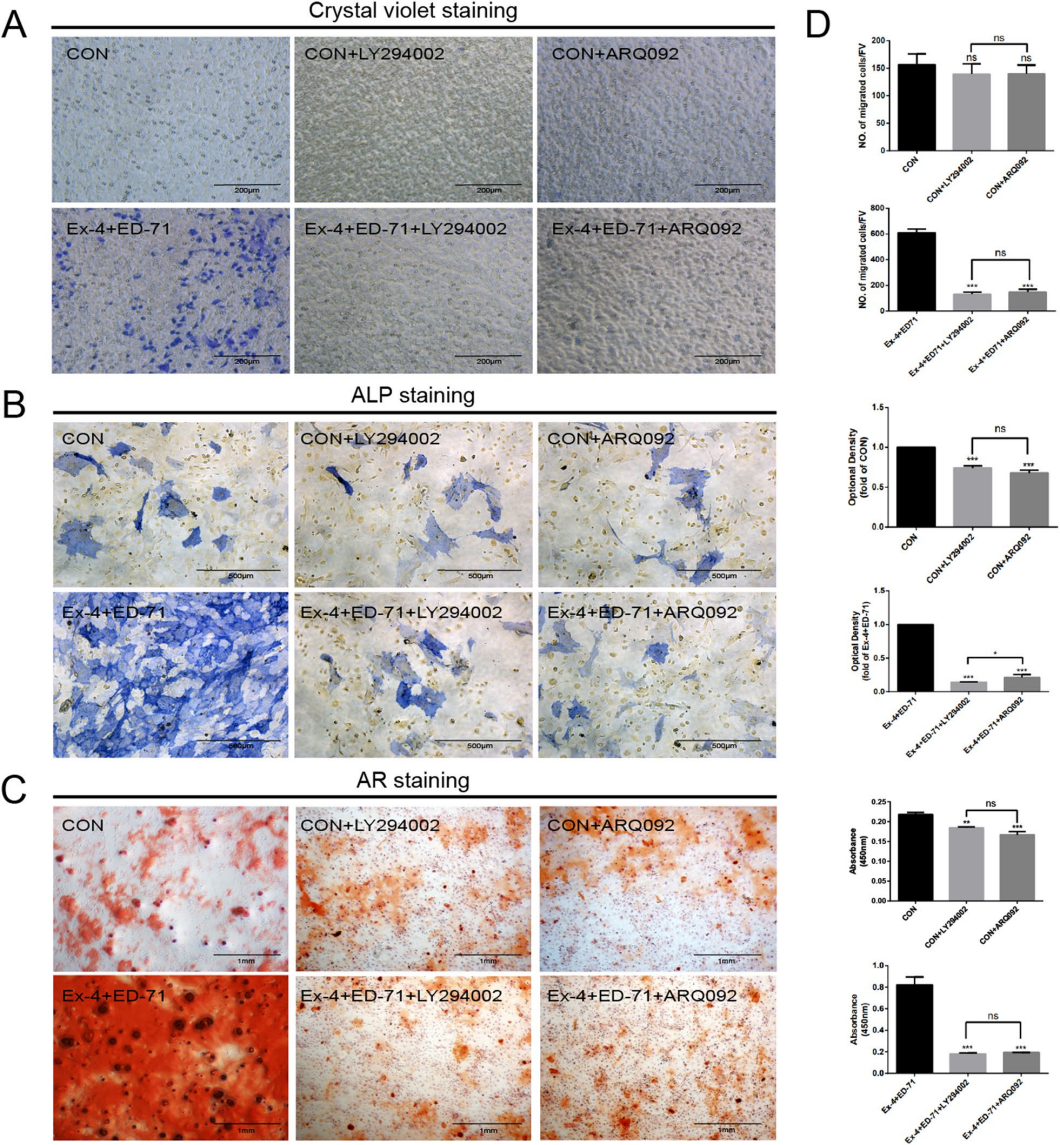
The combination of Ex-4 and ED-71 regulates macrophages through PI3K/AKT signaling pathway to promote osteogenic differentiation of BMSCs. **A** Crystal violet staining showed that the migration of BMSCs was inhibited after adding the small molecule inhibitors LY294002 and ARQ092. **B** and **C** ALP staining (**B**) and AR staining (**C**) showed that the osteogenic differentiation of BMSCs was inhibited after the addition of LY294002 and ARQ092. **D** The results of crystal violet, ALP and AR staining were quantitatively evaluated. *p < 0.05, **p < 0.01, ***p < 0.001. *CON* control, *Ex-4* exendin-4, *ED-71* eldecalcitol, *Ex-4* + *ED-71* exendin-4 + eldecalcitol, *LY294002* PI3K inhibitor, *ARQ092* AKT inhibitor, *ALP* alkaline phosphatase, *AR* Alizarin red

The effect of drugs on promoting migration and osteogenic differentiation of BMSCs are lowered significantly by small molecule inhibitors (LY294002 or ARQ092) as demonstrated through crystal violet staining (Fig. 6A), ALP staining (Fig. 6B) and AR staining (Fig. 6C). In detail, the groups administered LY294002 or ARQ092 alone had little effect on migration and osteogenic differentiation of BMSCs compared with the CON group. After macrophages were stimulated by the combination of Ex-4 and ED-71, the migration and osteogenic differentiation of BMSCs were significantly enhanced. However, it was found that the area of positive expression in the combination administration group was significantly reduced after treatment with LY294002 and ARQ092, illustrating that the migration and osteogenic differentiation of BMSCs were significantly weakened after adding LY294002 or ARQ092 in the presence of Ex-4 and ED-71. Therefore, we concluded that the combination of Ex-4 and ED-71 regulates macrophages through the PI3K/AKT signaling pathway to promote the migration and osteogenic differentiation of BMSCs.

### Ex-4 and ED-71 synergistically promote osteogenic differentiation of BMSCs through M2 macrophages polarization via the PI3K/AKT pathway

**Fig. 7:**
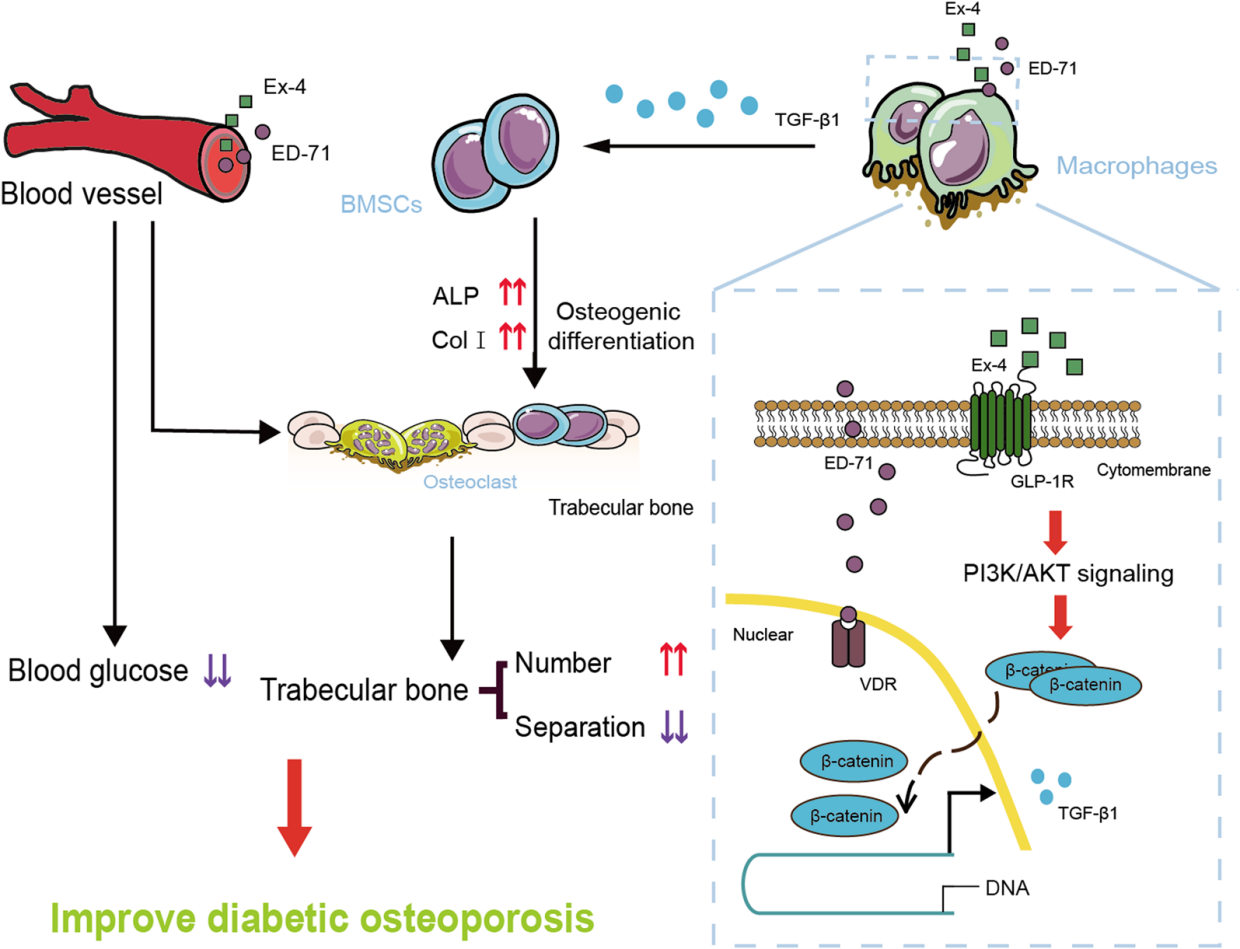
Ex-4 and ED-71 synergistically promote osteogenic differentiation of BMSCs through M2 macrophages polarization via PI3K/AKT pathway. Ex-4 and ED-71 synergistically activate the PI3K/AKT signaling pathway to polarize macrophages into M2, thereby producing a large amount of TGF-β1. Subsequently, BMSCs migrate and undergo osteogenic differentiation under the action of TGF-β1. In addition, the combination of these two drugs can synergistically reduce blood glucose levels to indirectly improve diabetic osteoporosis. *Ex-4* Exendin-4, *ED-71* Eldecalcitol, *GLP-1R* glucagon like peptide-1 receptor, *VDR* vitamin D receptor, *PI3K* phosphatidylinositol 3-kinase, *AKT* protein kinase B, *TGF-β1* Transforming growth factor-β1, *BMSCs* bone marrow mesenchymal stem cells, *ALP* alkaline phosphatase, *Col I* collagen I

Thus, we demonstrated that Ex-4 and ED-71 synergistically activate the PI3K/AKT pathway to polarize macrophages into M2, thereby causing osteogenesis differentiation of BMSCs (Fig. 7). In macrophages, Ex-4 and ED-71 bind to their cognate receptors and stimulate the transfer of β-catenin to the nucleus through the PI3K/AKT pathway, resulting in alterations to gene transcription. This process can polarize macrophages into M2 and stimulate the production of TGF-β1. Subsequently, BMSCs migrate and undergo osteogenic differentiation under the action of TGF-β1. In addition, the combined application of Ex-4 and ED-71 significantly reduced blood glucose levels, which may be an indirect way to improve diabetic osteoporosis.

## Discussion

The cause of diabetic osteoporosis can be divided into two categories. On one hand, the osteogenic differentiation of BMSCs is inhibited [16], and the absorption function of osteoclasts is relatively enhanced in patients with diabetic osteoporosis [17, 18]. The dynamic balance of bone remodeling is upset. In severe cases, pathological fractures may occur. On the other hand, the compromise of bone vasculatures is indirect factors leading to diabetic osteoporosis [19]. Therefore, reducing blood glucose levels and improving bone microstructure have become an important problem to be solved. Previous studies have shown that Ex-4 can significantly improve β-cell function to regulate blood glucose levels in diabetic patients [20]; however, the bone mineral densities were not affected significantly [21]. Conversely, ED-71, an analog of active vitamin D_3_, can prevent the decrease of bone biomechanical strength, improve bone mineral density and prevent diabetes-induced deterioration of trabecular microstructure in diabetic rats [22]. Therefore, we explored the possible synergistic relationship between Ex-4 and ED-71 in regulating blood glucose levels and inducing the osteogenic differentiation of BMSCs.

In this study, db/db mice treated with the combination of Ex-4 and ED-71 showed significantly enhanced bone formation and upregulated expression of related osteogenic factors. ALP, a factor of early osteoblast differentiation, was increased slightly in Ex-4 or ED-71 groups, but was significantly increased in the combination group, suggesting that Ex-4 and ED-71 could synergistically induce early osteoblast differentiation. Col I, a marker of advanced osteogenic differentiation, was significantly increased in mice treated with the combination of Ex-4 and ED-71, suggesting that these two drugs could rescue collagen fibers from damage caused by high glucose levels and play a synergistic role in the process of late osteogenesis. Thus, Ex-4 and ED-71 could synergistically promote bone formation in diabetic osteoporosis mice.

Furthermore, it was found that macrophages are increasingly polarized into M2 after treatment with ED-71 in db/db mice, and the combined application of Ex-4 and ED-71 could further increase this differentiation. It has been shown that communication between macrophages and BMSCs plays a role in bone regeneration. In particular, M2 macrophages can significantly promote osteogenesis mediated by BMSCs [23]. Our findings were consistent with this conclusion. In the co-culture system, macrophages treated with the combination of Ex-4 and ED-71 could induce more osteogenic differentiation of BMSCs. This result may be related to the production of more chemokines (TGF-β1) by M2 macrophages. It has been reported that TGF-β1 could regulate the migration and recruitment of BMSCs to participate in tissue repair [24]. Specifically, TGF-β1 recruits BMSCs to sites of active bone remodeling through the canonical pSmad2/3 signaling pathway [25]. In addition, TGF-β1 is also a powerful enhancer of the osteoinductive activity of BMP-2 [26]. Low doses of TGF-β1 can activate Smad3, promote its binding to the BMP promoter and up-regulate the expression of BMP-2 in BMSCs, thereby promoting the differentiation of osteoblasts. On the contrary, high doses of TGF-β1 inhibited the osteogenic differentiation of BMSCs and impaired bone regeneration [27].

Multiple signaling pathways can regulate the polarization of macrophages into different phenotypes [28], among which PI3K/AKT is one of the most important pathways for the polarization of M2 macrophages [29]. Many studies have shown that Ex-4 and vitamin D can promote M2 macrophage polarization [30, 31]. However, it was not clear whether the potential mechanism of the combination of Ex-4 and ED-71 on macrophage polarization involves PI3K/AKT. In this study, it was found that after RAW264.7 cells were co-treated with Ex-4 and ED-71, the polarization of M2 macrophages was significantly increased, the activation of PI3K/AKT signaling pathway was amplified, and the secretion of TGF-β1 was significantly increased compared with RAW264.7 cells treated with Ex-4 or ED-71 alone. This may be due to the fact that during the activation of PI3K/AKT, AKT increases the stability of β-catenin, and causes its accumulation and translocation into the nucleus [32, 33], thus promoting the polarization of M2 macrophages [15, 34]. In this process, the transduction of the β-catenin signal can promote the production of active TGF-β1 in macrophages [35].

In addition to these relatively direct mechanisms, we believe that the synergistic reduction of blood glucose also contributes to the impact of Ex-4 and ED-71 on the promotion of bone formation. Vitamin D supplementation can effectively reduce the risk of developing T2DM [36]. Several mechanisms explaining this link between vitamin D and glucose regulation have been identified. For instance, it has been reported that vitamin D can down-regulate the expression of caspase-3 to increase cell survival, thereby increasing the secretion of insulin from β-cells. Moreover, vitamin D can down-regulate the RNA component of telomerase (Terc) to increase the survival and retention of function of β-cells. Vitamin D can increase the release of prolactin-releasing hormone (Prlh), which increases insulin secretion by up-regulating prolactin levels [37]. Our results augment these mechanistic studies. In db/db mice, an animal model of T2DM, treatment with ED-71, a vitamin D analog, resulted in significant improvement in blood glucose levels, which was similar to the previously reported effects of vitamin D and other analogs, such as calcipotriol [38]. Interestingly, our study demonstrated for the first time that the combination of Ex-4 and ED-71 can lower blood glucose levels, and that this lowering was more effective in combination than with either drug used alone. The decrease in blood glucose likely resulted in decreases in the accumulation of advanced glycation end-products (AGEs) and reactive oxygen species (ROS), which may explain the reduction in damage to bone tissue. This may be one of the mechanisms by which the combination treatment synergistically improves diabetic osteoporosis.

Importantly, treatment with the drug combination was well tolerated by the mice. However, it must be noted that the most common adverse drug reaction of ED-71 is hypercalcemia [39], and we found that blood calcium levels were significantly increased in ED-71 and combination groups. There was no significant difference in blood calcium levels between the ED-71 monotherapy and combination therapy groups, which means that the two drugs did not have a synergistic effect on this potentially negative outcome. If the combination treatment of Ex-4 and ED-71 is explored for use in clinic, however, attention must be paid to monitoring blood calcium levels of patients to prevent a series of complications, including hypercalcemia.

There is a limitation in this subject. Although Ex-4 and ED-71 were only applied to RAW264.7 in the co-culture system, BMSCs were directly exposed to these two drugs simultaneously due to the permeability of the transwell membrane. The direct effect of drugs on BMSCs was not fully considered in this study. And this will be our new research direction.

## Conclusion

We investigated the bone repair effect of Ex-4 combined with ED-71 in the treatment of diabetic osteoporosis. This bone repair effect may be related to the communication between macrophages and BMSCs. Ex-4 and ED-71 synergistically promote osteogenic differentiation of BMSCs through M2 macrophage polarization via the PI3K/AKT pathway. In addition, combination treatment is associated with a synergistic decrease in blood glucose levels. Therefore, the combined application of Ex-4 and ED-71 may be a potential treatment for diabetic osteoporosis.

## Data Availability

The datasets used and/or analyzed during the current study are available from the corresponding author on reasonable request.

## Abbreviations

Ex-4: Exendin-4
ED-71: Eldecalcitol
BMSCs: Bone marrow mesenchymal stem cells
TGF-β1: Transforming growth factor-β1
PI3K/AKT: Phosphatidylinositol 3 kinase/protein kinase B
ALP: Alkaline phosphatase
Col I: Collagen I
